# Diagnostic Utility of Surface Electromyography for Identifying Muscles Affected by Myofascial Trigger Points: A Scoping Review

**DOI:** 10.3390/biomedicines14061406

**Published:** 2026-06-22

**Authors:** Jakub Matuska, Ryszard Śliwiński, Jędrzej Pepliński, Wiktoria Frącz, Clara Leśniak, Elżbieta Skorupska, Manel M. Santafé

**Affiliations:** 1 Doctoral School, Poznan University of Medical Sciences, 61-702 Poznan, Poland; 2 Unit of Histology and Neurobiology, Department of Basic Medical Sciences, Faculty of Medicine and Health Sciences, Rovira I Virgili University, 43003 Reus, Spain; 3 The Student Scientific Society, Poznan University of Medical Sciences, 61-702 Poznan, Poland; 4 Faculty of Health Sciences, University of Zaragoza, 50009 Zaragoza, Spain; 5 Department of Physiotherapy, Poznan University of Medical Sciences, 61-702 Poznan, Poland

**Keywords:** myofascial pain, motor unit recruitment, electrophysiology, chronic musculoskeletal pain, referred pain, neuromuscular control

## Abstract

**Background**: The diagnostic value of surface electromyography (sEMG) for identifying muscles affected by myofascial trigger points (TrPs) remains controversial. However, advances in pain neurophysiology and discussions regarding TrPs within the International Classification of Diseases (ICD-11) have renewed interest in objective diagnostic approaches. **Objective**: To synthesize current evidence on the diagnostic utility of sEMG for detecting TrP-related muscle alterations across different electromyographic signal analysis domains. **Methods**: A scoping review was conducted following JBI guidance and PRISMA-ScR guidelines. PubMed, Scopus, Web of Science, CINAHL and Cochrane were searched for studies involving adults with symptomatic or asymptomatic TrPs, myofascial pain syndrome, or TrP-related referred pain. Fifteen studies met the inclusion criteria. Analyses included amplitude-, frequency-, time–frequency-, and spatial-domain sEMG parameters. **Results**: Muscles affected by TrPs showed increased resting electromyographic activity and reduced activation during maximal voluntary contraction in several studies. Frequency domain analyses indicated changes in median frequency and muscle fatigue index, whereas time–frequency analyses suggested redistribution of sEMG signal energy toward lower-frequency components or altered spectral power during experimentally provoked referred pain. Spatial analyses revealed altered activation patterns, although these findings did not consistently correspond with TrP anatomical locations. Overall, the limited number of studies assessing diagnostic sensitivity and specificity prevents firm conclusions. **Conclusions**: sEMG may be useful as a non-invasive complementary tool for functional assessment and monitoring of TrP-related muscle dysfunction. However, current evidence does not support its use as a standalone diagnostic method. Time–frequency, machine learning-supported and spatial analyses appear promising for future clinical research, but standardized protocols and external validation are required before clinical diagnostic criteria can be proposed.

## 1. Introduction

For many years, myofascial pain syndrome (MPS), associated with myofascial trigger points (TrPs), has been controversial and not uniformly accepted [1]. Despite this, TrPs have been widely used in clinical practice by physicians and physiotherapists. According to Travell and Simons’ criteria, further supported by an international consensus, TrP diagnosis is based on a cluster of clinical features, including a palpable taut band, a hypersensitive spot, and referred pain. Clinically, TrPs are divided into active points, which reproduce the patient’s symptoms, and latent points, which are painful only on palpation [2,3]. The perception began to shift with the discussion on chronic pain categories in the 11th revision of the International Classification of Diseases (ICD-11) [4,5,6]. With ICD-11 (adopted in 2019; in effect from 2022), chronic pain is represented by dedicated categories (MG30), increasing the visibility of chronic pain conditions and their subtypes [7,8]. However, the placement of myofascial pain/TrPs within chronic primary versus chronic secondary musculoskeletal pain remains debated. This still requires more evidence, especially regarding motor, autonomic, and sensory components [4,9]. Despite the lack of a clear classification in ICD-11, TrPs remain an important element in the diagnosis and treatment of myofascial pain in clinical practice [10].

An increasing number of studies highlight the presence of TrPs in patients with chronic pain, as well as the effectiveness of various therapeutic approaches [11,12,13]. So far, one of the main challenges with TrP diagnosis is that it is still based on manual palpation, which may lack objectivity [14,15]. Therefore, there is a strong need for the development of new diagnostic tools. Attempts were made to adapt already existing methods for TrP assessment. These include ultrasonography (US) [16,17], magnetic resonance imaging (MRI) [18,19], thermography [20,21], needle electromyography (nEMG) [22], surface electromyography (sEMG) [23,24,25,26,27,28], and US with elastography [17,29]. Each method has strengths and limitations. US depends heavily on operator skill, MRI is expensive, thermography has mostly non-standardized protocols, US with elastography does not yet appear decisive, and nEMG, while well-supported by evidence, is invasive [17,18,19,21,22]. However, increased motor endplate noise detected by nEMG is considered an important electrophysiological feature associated with TrPs. Its presence reflects abnormal spontaneous electrical activity related to dysfunctional motor endplates [22,30,31]. This is consistent with Simons’ integrated hypothesis, which associates TrPs with excessive acetylcholine release, abnormal endplate activity, and sustained local sarcomere contraction. The simultaneous release of multiple synaptic vesicles from a nerve terminal generates an endplate potential, which subsequently elicits muscle fibre action potentials [32]. The spikes detected by electromyography correspond to action potentials [33]. Consequently, the excessive acetylcholine release associated with TrPs may be reflected electromyographically as an increased spike activity, which can be detected using sEMG.

While nEMG may provide diagnostically relevant information, sEMG offers a non-invasive and more accessible method for assessing muscle activation in clinical and research settings. Because sEMG does not record endplate activity, its diagnostic relevance for TrPs should be interpreted through changes in muscle activation patterns, motor control, and signal features associated with TrP-affected muscles. This is important because neurophysiological dysfunction is increasingly recognized as a component of musculoskeletal pain conditions, especially when symptoms involve altered motor control or functional impairment that is not fully explained by structural findings alone [5,34]. Such changes may reflect pain-related adaptations in motor unit recruitment, spinal excitability, proprioceptive feedback, and coordination between muscle groups [35]. In pelvic and lower-limb conditions, TrPs, referred pain or altered afferent input may influence neuromuscular control beyond the primary nociceptive site [26,36].

In sEMG analysis, several signal components are often analyzed, such as: amplitude, frequency, time–frequency, and spatial characteristics [37,38]. These components may reveal muscle activation patterns associated with TrP-related dysfunction. Many studies using sEMG have reported findings like altered motor patterns, reduced muscle activity, fatigue, and weakness in muscles affected by TrPs [25,28,39,40]. Existing reviews have addressed the diagnosis of TrPs more broadly, but not the specific diagnostic utility of sEMG [17,18,21,41]. Thus, the overall accuracy of sEMG for TrP diagnosis has not yet been summarized.

The PCC framework was used a priori to define the scope of this review and to justify the need for mapping existing evidence on the diagnostic utility and potential of sEMG in TrPs. Therefore, the aim of this review is to summarize the current evidence on the potential diagnostic relevance of sEMG in identifying TrP-affected muscles, with particular attention to the different signal analysis domains.

## 2. Materials and Methods

This scoping review was conducted in accordance with the methodology for scoping reviews (PRISMA-ScR), as detailed in Supplementary Materials (File S1: Table S1: PRISMA-ScR checklist) [42]. No review protocol was registered prior to conducting this scoping review. An internal protocol was developed a priori for use by the review team (File S2: Review Protocol). The need for the review was evaluated using the PCC framework (Population, Concept, Context): P—adult individuals with muscles affected by TrPs; C—assessment of muscle properties using sEMG, with a focus on its potential diagnostic utility in identifying TrPs; C—clinical, laboratory, or experimental research in which sEMG was used to assess occurrence of TrPs [43]. We conducted a critical appraisal of the included studies to strengthen the transparency and interpretability of the findings. It was performed using JBI critical appraisal tools selected according to study design [44,45,46,47] and the results were used to support interpretation of the evidence rather than to determine study eligibility (File S1: Figure S1A–C: Critical Appraisal).

### 2.1. Search Methods

The following databases were searched: PubMed, Scopus, Web of Science, CINAHL and Cochrane. The initial search was conducted between 20 April and 25 April 2024, and an update search was conducted on 12 May 2026. The update was conducted to capture newly published studies. Search terms combined electromyography/sEMG with myofascial pain and TrPs. The search strategy was initially developed in PubMed using MeSH Terms and title/abstract. Next it was adapted to the other databases. Additionally, the “Similar Articles” feature was used to identify further relevant studies. Records from the first and “Similar Articles” feature were combined before duplicate removal. Table 1 reports the search strategy.

### 2.2. Inclusion and Exclusion Criteria

Three reviewers independently assessed the eligibility of each study between 25 and 30 May 2024. For records identified in the second search, inclusion and exclusion criteria were verified on 13 May 2026. Consensus among all three reviewers was required for study inclusion. In cases where agreement could not be reached through discussion, the final decision was made by the supervising author. Because this was a scoping review, a separate healthy control group was not required in every study. Each included study had to provide a meaningful comparator or reference condition to interpret findings in relation to TrPs. The eligibility criteria are summarized in Table 2.

### 2.3. Data Extraction

Citations were retrieved from relevant databases and imported into Rayyan.ai (Rayyan Systems, Inc., Cambridge, MA, USA) [48]. Duplicate records were automatically detected by the software and subsequently verified by three reviewers. Titles and abstracts were screened independently according to defined inclusion and exclusion criteria. Full-text articles were then assessed for eligibility. Data extraction was performed independently by three reviewers using a standardized form developed for the purposes of this review. Any discrepancies were discussed among the reviewers; if consensus was not reached, the supervising author made the final decision. Extracted variables included study characteristics, participant demographics, details of TrPs diagnosis, comparator/reference condition, sEMG parameters, methodological features, and reported outcomes. The collected data were categorized into three sections: general characteristics, technical aspects, and sEMG-related results.

## 3. Results

**Figure 1:**
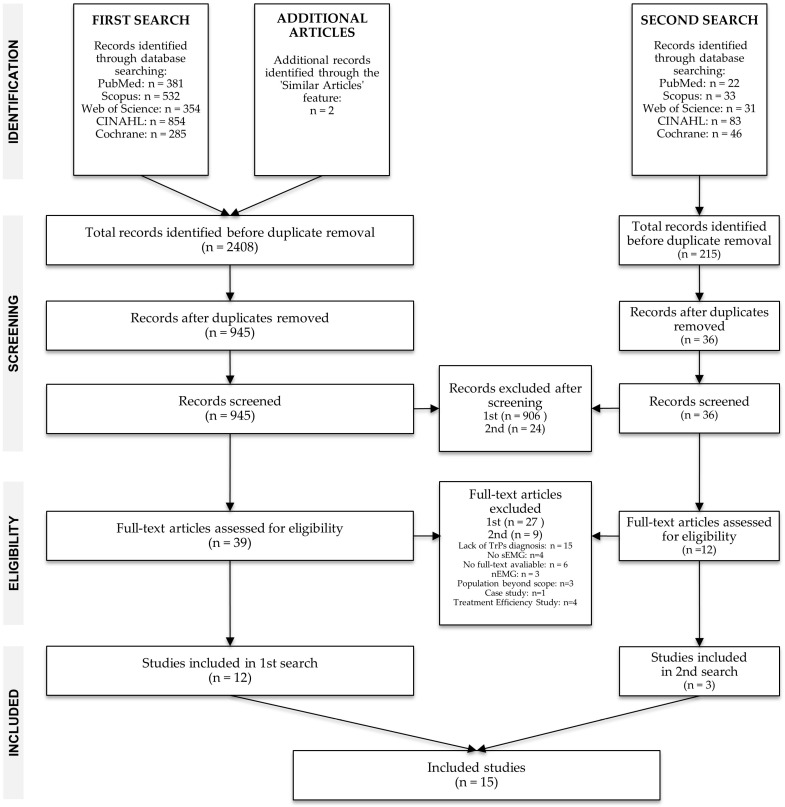
A PRISMA-ScR flow diagram illustrating the study selection process. Records were identified through the first database search, the second search and the “Similar Articles” feature. After duplicate removal, the records were screened and full-text articles were assessed according to the eligibility criteria. Fifteen studies met the inclusion criteria and were included in the final scoping review. The first search was conducted in April 2024 and second in May 2026.

The database searches identified 2621 records including 2406 records from the first and 215 records from the second search. After duplicate removal, 943 records were screened. Additionally two articles were included due to the “Similar Articles” feature. Full-text records were then assessed with the eligibility criteria. Ultimately, 15 studies were included in this review (Figure 1).

The critical appraisal of the included studies is summarized in Supplementary Materials (File S1: Figure S1A–C: Critical Appraisal) and supports the cautious interpretation of the current evidence.

### 3.1. General Characteristics of the Included Studies

Most studies examined sEMG patterns in muscles with TrPs or MPS in comparison with a healthy/asymptomatic/latent TrPs group or other conditions. The anatomical regions investigated varied across studies. Sample sizes ranged from 12 to 120 participants, predominantly young to middle-aged adults with uneven gender distribution. Diagnostic criteria varied, most commonly including taut band (12/15 studies), hyperirritable spot (12/15), referred pain (8/15), and local twitch response (4/15). Among the nine studies that differentiated active from latent TrPs, five used familiar symptom elicitation as a criterion. Some studies focused on MPS diagnosis, while one used the Research Diagnostic Criteria for Temporomandibular Disorders (RDC/TMD) Axis I. These results are shown in Table 3.

### 3.2. Technical Aspects of sEMG Procedures

As shown in Table 4, sEMG properties varied among the included studies. Most studies placed two electrodes over muscle bellies, following SENIAM guidelines. Three studies [28,40,56] used high-density electrode grids placed over the upper trapezius muscle. One study [55] placed electrodes along the taut band. Signal collection properties differed between studies, with commonly reported band-pass filters ranging from 10 Hz to 500 Hz. Furthermore, sampling rates varied from 1200 Hz to 5000 Hz.

Only three studies reported signal normalization, but it remained heterogeneous. Gemmell and Bagust [51] normalized RMS amplitude to the mean value recorded during shoulder shrugs, Rahmati et al. [53]. normalized EMG amplitudes to MVC values, and Lu et al. [56] applied Z-score standardization.

### 3.3. sEMG Results Across Different Domains of Analysis

The included studies were grouped according to the sEMG analysis domain: amplitude domain (*n* = 8), frequency domain (*n* = 1), time–frequency (*n* = 2), combined time–frequency/amplitude/frequency domain (*n* = 1), and HD-sEMG domain (*n* = 3). Machine learning-based classification was reported in two studies, using MSWEV-derived features in Lin et al. [27] and HD-sEMG contraction segments in Lu et al. [56].

Diagnostic or classification metrics were reported in four studies. Manfredini et al. [49] reported sensitivity/specificity for clenching tasks (77.8–91.7%/76.7–86.7%) and at rest (43.5–52.2%/27.8–55.6%). Jiang et al. [55] reported sensitivity/specificity for visual MSWEV classification (53.85%/83.33%). Lin et al. [27]. reported classification accuracy of 77% with template matching and 60% with K-means clustering, with consistency of 87% in control group and 93% in MPS. Lu et al. [56] reported subject-level ROC-AUC/accuracy of 0.940/0.960 for full-group pre-intervention one-dimensional convolutional neural network (1D CNN) HD-sEMG classification and 0.916/0.930 for latent-TrPs combined pre/post signals.

The main findings of EMG analyses (Table 5) from the included studies are presented below, divided according to the type of EMG analysis: amplitude domain, frequency domain, time–frequency domain, and spatial EMG distribution.

#### 3.3.1. Amplitude Domain Analysis

Amplitude domain outcomes were extracted from recordings at rest, during MVC, or during task-specific contractions (Table 5). Four studies reported higher resting sEMG amplitude in muscles with TrPs compared with reference conditions [23,24,25,52]. During MVC or clenching tasks, some studies reported lower amplitudes in myofascial pain groups than in healthy controls [25,49,52], whereas clenching findings in Zieliński et al. [23] were parameter- and comparison-dependent. Manfredini et al. [49] reported no significant resting amplitude difference and found acceptable diagnostic metrics mainly for clenching tasks, whereas resting measures had high false-positive rates. These findings suggest that amplitude-domain sEMG may be informative at the group level, but it is not specific to TrPs.

As shown in Table 5, the diagnostic or classification-oriented sEMG outcomes were explored in four studies. Two teams focused on differentiating between clinical conditions, while a third examined electrophysiological distinctions between latent and active TrPs. Zieliński et al. (2021) [23] compared sEMG patterns in TMD patients and those with trapezius TrPs, concluding that while both conditions alter electromyographic activity in masticatory muscles, further research is needed to define specific diagnostic parameters. Huber and Lisiński (2017) [25] emphasized the need for multiple neurophysiological tests, proposing that the trapezius muscle is key in myofascial pain. At the same time, the abductor pollicis brevis may help differentiate symptoms caused by TrP-affected muscles from nerve-root conflicts. Most studies did not distinguish between latent and active TrPs. Only Gemmell and Bagust (2009) [51] noted a potential trend toward lower RMS in active TrPs than latent ones, though the difference was not statistically significant. Rahmati et al. [53] reported that peak EMG amplitude and RMS did not differ significantly between an active TrP group and healthy controls during shoulder abduction. However, rise time was prolonged in the TrP group.

In summary, several studies reported higher resting sEMG amplitude in muscles with TrPs, whereas reduced amplitude during MVC was less consistent and depended on the task and comparison group. Moreover, diagnostic accuracy was acceptable mainly during MVC tasks, while resting measures showed high false-positive rates. One study reported that not amplitude but the time from EMG onset to peak EMG amplitude differed between TrPs and the healthy group.

#### 3.3.2. Frequency Domain Analysis

In the included studies, median frequency (MDF), muscle fatigue index (MFI), and power spectral density (PSD) were calculated based on the signal acquired during MVC or specific experimental/provocation tasks (see Table 5). Yu and Kim [54] found higher MDF and MFI values in muscles with active TrPs compared with healthy controls, suggesting potential differences. By contrast, Jiang et al. [55] observed lower MDF in MFP patients during some phases of the arm-lifting phase. However, they concluded that MDF alone lacks sufficient diagnostic specificity, emphasizing the need for multi-parameter approaches. Konieczny et al. [26] reported that higher PSD was observed in the group with experimentally provoked referred pain.

In summary, the analyzed studies indicate that MDF, MFI, and PSD differ between TrP-related and reference conditions, but these measures reflect different physiological constructs. Conventional frequency-domain parameters remain nonspecific.

#### 3.3.3. Time–Frequency Analysis

Time–frequency analysis using MSWEV was applied by the same research team in Jiang et al. (2013) [55] and Lin et al. (2018) [27] to characterize neuromuscular changes in MPS (see Table 5). Jiang et al. [55] reported that healthy controls showed relatively greater high-frequency energy, whereas MPS patients showed relatively greater low-frequency band energy. Jiang et al. [55] also reported sensitivity and specificity values for MSWEV visual classification. Lin et al. [27] subsequently used machine learning-based MSWEV patterns and reported accuracy and consistency. Furthermore, Konieczny et al. [26] employed short-time Fourier transform (STFT), demonstrating that the occurrence of referred pain was associated with altered bioelectrical muscle activity.

In summary, MSWEV-based time–frequency analyses showed altered spectral profiles in MPS, whereas STFT captured transient spectral changes during experimentally elicited TrP-related referred pain.

#### 3.3.4. High-Density sEMG Analysis

As shown in Table 5, two studies investigated spatial HDsEMG patterns in relation to TrPs. Both studies by Barbero et al. [28,56] identified consistent innervation zones (IZs) in the upper trapezius, finding no spatial overlap between IZ and TrP locations despite their proximal arrangement. In shoulder elevation tasks, subjects with TrPs exhibited a caudal shift in peak EMG amplitude distribution compared to controls, suggesting altered motor adaptation or recruitment patterns. However, no direct correlation was found between EMG amplitude peaks and TrP locations. Lu et al. [56] used HD-sEMG recordings from the upper trapezius and applied a 1D CNN to distinguish participants with TrPs and healthy controls. The authors obtained the best subject-level classification using pre-intervention contraction segments.

In summary, spatial EMG studies showed no overlap between IZs and TrPs, despite their proximity. A caudal shift in EMG amplitude was observed in TrP subjects, without direct spatial correspondence to TrP locations. Furthermore, HD-sEMG contraction segments may contain discriminative information for TrP classification with machine learning.

## 4. Discussion

Overall, the available evidence supports a narrow conclusion: sEMG can detect group-level alterations in muscles with TrPs or TrP-related referred pain, but current data do not justify its use as a standalone diagnostic test. Only four studies reported diagnostic or classification metrics, and none provided external validation. Amplitude and conventional frequency-domain measures were particularly nonspecific, whereas time–frequency, HD-sEMG, and automated classification approaches require replication under standardized protocols.

Across the included studies, TrP-affected muscles most frequently exhibited altered amplitude parameters as increased at rest activity and reduced amplitude during MVC. In the frequency domain, Yu and Kim [54] reported higher MDF/MFI, whereas Jiang et al. [55] reported selected lower MDF values. Next, MSWEV-based studies suggested a shift in the energy toward lower-frequency components for patients with MPS. Furthermore, in the time–frequency domain Konieczny et al. [26] showed altered spectral power during provoked referred pain. Among studies utilizing HDsEMG, spatial analyses indicated altered distribution of upper trapezius EMG activity, although no consistent overlap was observed between TrP locations and IZs. Moreover, Lu et al. [56] suggested that HD-sEMG contraction segments may provide discriminative information for TrPs when analyzed with a CNN.

Each analytical approach showed both advantages and limitations. The following sections discuss neurophysiological interpretation, diagnostic relevance by sEMG domain, and the current limitations requiring further investigation.

### 4.1. Neurophysiological Mechanisms Underlying sEMG Alterations in TrP-Affected Muscles

Across the included studies, muscles affected by TrPs showed a recurring pattern of altered but not uniform sEMG behaviour. TrPs are commonly discussed in relation to spontaneous electrical activity and motor endplate dysfunction, which have been described in human and animal models [22,31,57]. However, the connection between these local phenomena and a signal recorded from the skin is not direct. Surface electromyography does not record spontaneous endplate activity as nEMG does. It represents the spatial and temporal summation of motor unit action potentials, after those potentials have passed through the volume conductor [37]. For this reason, the amplitude and spectral content of sEMG are shaped not only by recruitment and discharge behaviour, but also by tissue geometry, fibre conduction properties, electrode placement, and crosstalk. In fact, sEMG should be read as an indirect sign of muscle activation or neural drive, not as a marker of endplate activity [37,58].

From a neurophysiological point of view, the most plausible explanation between TrPs and altered sEMG is a change in motor output driven by nociceptive, mechanosensitive, and proprioceptive input. The nociceptive component is mediated by small-diameter group III and IV muscle afferents, which respond to noxious mechanical and chemical stimuli. It provides a substrate for pain-related motor adaptation and referred pain [59,60,61]. A proprioceptive component is also possible, but it should be presented more cautiously. Electrophysiological human study and experimental animal models have shown enhanced H-reflex activity at TrPs and a close anatomical relationship between TrPs and muscle spindles [62,63]. More recent data further suggest that abnormal spontaneous potentials recorded at TrPs can be modulated by stretch and by pharmacological manipulation of spindle activity [64]. Thus, enhanced H-reflex indicates increased spinal motoneuron excitability [65]. Taken together, these findings support the possibility that altered Ia/II spindle-related feedback may contribute to reflex modulation of the motoneuron pool, whereas group III/IV input is more likely to mediate nociceptive drive, referred pain, and pain-related motor adaptation.

In active TrPs, ongoing nociceptive input may change excitability within spinal reflex pathways and supraspinal motor networks [59,66]. The motor response may be seen as a change in recruitment, discharge organization, inhibition between agonists and antagonists, or redistribution of activity within a muscle and across neighbouring muscles. In this sense, TrPs may influence sEMG because local nociceptive and mechanical disturbances can change the net output of the motoneuron pool [37,59,66,67].

This interpretation is also for considering both active and latent TrPs. Active TrPs are associated with spontaneous pain or pain recognized by the patient, whereas latent TrPs do not produce spontaneous symptoms [3]. Nevertheless, the absence of spontaneous pain does not necessarily mean that the muscle behaves normally. In both forms, local mechanical and biochemical changes within the taut band may increase mechanical sensitivity and modify afferent input from the affected muscle [22,68]. This may include muscle spindle-related feedback and large-diameter muscle afferent pathways, affecting spinal reflex excitability and the output of the alpha motoneuron pool [62,63,64,69]. This gives a reasonable explanation for why some studies reported changes in sEMG parameters despite the absence of pain.

The findings obtained from muscles located within a referred pain area should be discussed separately. Konieczny et al. [26] reported spectral changes in muscles located within the referred pain distribution after noxious stimulation of a TrP. These changes do not necessarily imply local pathology in the recorded muscle. A possible explanation is that stimulation of the gluteus minimus activated muscle nociceptors and segmental spinal circuits that also receive input from thigh muscles in the referred pain area. Through this convergence, referred pain may be accompanied by a short-lived change in motor output in muscles that were not directly stimulated [70,71]. This interpretation is also compatible with the observations of Skorupska et al., who showed that noxiously provoked referred pain can be accompanied by abnormal autonomic reactivity within the referred pain zone [72]. Furthermore, Bank et al. did not examine muscles within referred pain areas, but their review supports the concept that experimentally induced pain can induce rapid changes in motor output beyond the directly painful tissue [59]. In the present context, this makes it plausible that muscles located within the referred pain distribution may show transient motor changes during noxious TrP stimulation, particularly if they share segmental nociceptive or sensorimotor circuits with the stimulated muscle. Still, sEMG alone cannot determine whether these spectral effects arise from changes in recruitment, synchronization, conduction properties, or other sources of signal variability [37,73].

### 4.2. Diagnostic Potential of Surface EMG for TrPs

In this review, most of the included studies revealed differences in sEMG parameters between symptomatic and healthy participants. To provide a detailed understanding, the diagnostic relevance of each sEMG analysis domain is discussed separately.

#### 4.2.1. Amplitude Domain

The amplitude-based sEMG analysis was the most frequently used domain in the reviewed studies. Most of them reported increased resting muscle activity in the presence of TrPs, although neurobiological explanations were rarely provided [23,24,25]. In this context, changes in resting sEMG amplitude are more likely to reflect altered motor unit behaviour or pain-related motor adaptation than a direct marker of local TrP pathology. These findings are often discussed within the framework of the Integrated Hypothesis, which links motor endplate dysfunction to local contracture, ischemia, and peripheral sensitization [74]. However, sEMG amplitude is not specific to TrPs and may also be influenced by electrode placement, tissue thickness, contraction level, fatigue, anxiety, and other painful musculoskeletal conditions. Manfredini et al. [49] found no difference in resting amplitude and warned against false positives, likely due to a lack of clinically useful cut-off values for amplitude.

During maximal MVC, reduced signal amplitude was observed in TrP-affected muscles [24,25,49]. This may reflect altered motor unit recruitment or pain-related central inhibition, as nociceptive input is known to modulate neuromuscular control [75,76,77,78]. Only Manfredini and colleagues indicated acceptable sensitivity and specificity for the maximal clenching task [49]. However, comparing differences in MVC amplitude can lead to some pitfalls due to several factors like tissue thickness, electrode placement and individual muscle strength [79]. Furthermore, even normalization to MVC may lead to misinterpretation in comparing healthy to pain patients (e.g., with TrP-affected muscles). Some studies indicated that pain influences muscle activity, leading to unrepresentative results, as pain inhibits the ability to produce maximal muscle force [80,81,82].

Taken together, it seems that amplitude alone lacks diagnostic specificity. Zieliński et al. [23] reported overlapping EMG patterns in TMD and TrPs. Moreover, Lisiński and Huber [25] showed that similar amplitude differences also occur in radiculopathy and TrPs. The findings by Rahmati et al. [53] reinforce this limitation. Peak EMG amplitude and RMS during shoulder abduction did not distinguish active upper-trapezius TrPs from controls, whereas rise time differed between groups. Thus, amplitude-domain sEMG analysis may be more informative when interpreted alongside timing or kinematic measures, but it remains insufficient as a standalone diagnostic marker. This supports the need for integrated diagnostic protocols, combining amplitude with other neurophysiological tests to improve accuracy and reduce misdiagnosis of TrPs.

#### 4.2.2. Frequency Domain

The reviewed studies indicate that frequency domain analysis may capture changes in TrP-affected muscles, but the physiological interpretation differs between parameters. At first sight, these frequency-related findings may seem inconsistent, with higher MDF/MFI in some studies, lower-frequency MSWEV patterns in others, and higher-band activity during provoked referred pain. However, these outcomes were obtained with different signal descriptors, recording conditions, and experimental contexts. They may therefore reflect different aspects of motor unit behaviour.

Yu and Kim [54] reported higher MDF and MFI in active TrP-affected muscles compared with healthy controls, suggesting altered recruitment strategies and a possible greater contribution of type II muscle fibres. In contrast, Jiang et al. [55] reported lower MDF in patients with MPS than in healthy controls during selected phases of the arm-lifting task. Because frequency domain findings are based on a small number of studies and lack consistent diagnostic metrics, these parameters should currently be interpreted as exploratory rather than diagnostic. Thus, this domain remains nonspecific and may be influenced by contraction level, motor unit recruitment strategy, and muscle fatigue [37]. Further studies are needed before these measures can be considered distinctive markers of TrP-affected muscles.

Konieczny et al. [26] proposed a different approach and focused on PSD within the referred pain area during noxious stimulation of TrP-affected muscle. The authors reported higher PSD values in muscles located within the area of experimentally provoked referred pain. This suggests a relationship between provoked referred pain and altered bioelectrical activity. The trigger point network theory, in which key TrPs may contribute to activation of so-called satellite TrPs, was proposed by the authors as one possible hypothesis [3,26]. Those results are also in line with findings by Fernandez Carnero et al., who showed increased spontaneous electrical activity and sensitivity in a distant latent TrP located at the same segmental level after nociceptive stimulation of another latent TrP [83]. Another electrophysiological explanation was proposed in the previous paragraph (Section 4.1). Nonetheless, the observations of elevated PSD in muscles within the referred pain area by Konieczny et al. [26]. align with the search for potential objective signs associated with experimentally provoked TrP-related referred pain.

#### 4.2.3. Time–Frequency Domain

Two studies, by Jiang et al. [55] and Lin et al. [27]., utilized MSWEV to distinguish between patients with MPS/TrP-affected muscles and healthy controls. Jiang et al. [55] reported sensitivity and specificity for a visual MSWEV approach, whereas Lin et al. [27] extended the MSWEV approach using machine learning methods and reported moderate classification accuracy/consistency. Their results indicated a shift in the distribution of sEMG signal energy toward lower-frequency components in MPS, which may be associated with altered recruitment of low-threshold motor units [27,55]. This predominance of low-frequency energy in this group may reflect tonic, low-level muscle activity associated with protective motor strategies or altered motor control without implying direct changes in muscle fibre composition [36,66,67,84]. The moderate accuracy reported by Lin et al. [27] may be partly explained by the fact that latent TrPs can occur in asymptomatic individuals. If latent TrPs were not explicitly screened out, control group misclassification could reduce classification performance [85]. At the current stage, MSWEV should be viewed as a descriptor of altered muscle activation. Its diagnostic value will depend on whether the reported energy-distribution patterns can be replicated.

Konieczny et al. [26] used STFT analysis, which further supports the potential relevance of frequency-based measures. The authors showed that if referred pain was experimentally provoked, there was an increase in spectral energy visible on spectrograms in muscles within the referred pain area. Provoked pattern of referred pain was characteristic for TrPs localized in the gluteus minimus muscle [26]. This suggests that referred pain provoked from TrP-affected muscles may be associated with a distinct sEMG response pattern. However, the interpretation of spectrograms recorded during resting conditions is less established clinically than their use in dynamic or fatigue-related tasks [37,86,87]. Hence, this association requires further investigation in terms of symptom timing, pattern dynamics, replication in larger patient cohorts, and to determine whether such patterns can provide clinically meaningful diagnostic information, including sensitivity and specificity.

#### 4.2.4. High-Density sEMG

High-density sEMG showed partial alterations in the spatial distribution associated with TrPs. Barbero et al. [40] demonstrated a caudal shift in sEMG amplitude in subjects with TrP-affected trapezius. Nevertheless, the authors found no direct overlap between the IZ and TrPs [28]. This suggests that the observed pattern may reflect motor adaptation to pain rather than the presence of TrPs at a specific location [67]. Furthermore, the localization of TrPs through palpation is prone to error because it relies on the examiner’s subjective assessment and the patient’s variable pain perception [15]. Therefore, these spatial findings should be treated with caution. Moreover, Srbely et al. [88] pointed out insufficient data linking the location of the average rectified value to the location of TrPs. They also noted that small sample size and individual variability were likely to affect Barbero et al.’s findings [88]. Furthermore, the findings by Barbero et al. [28,40] are not in line with previous studies showing increased local bioelectrical activity within TrP-affected muscles in nEMG [31]. However, spatial sEMG findings are not directly comparable with intramuscular EMG because both methods capture signals at different physiological and anatomical levels. The absence of overlap between TrP location and IZs does not rule out local TrP-related electrophysiological phenomena. Therefore, HD-sEMG should currently be used with caution as a mapping tool for direct TrP localization.

Lu et al. [56] extended HD-sEMG analysis toward automated classification by applying a 1D CNN to contraction segments. The reported subject-level ROC-AUC and accuracy values were high. However, the authors did not identify a specific physiological sEMG alteration such as increased amplitude, frequency shift, or spatial displacement of peak activity. They showed that contraction segments contained class-discriminative information when analyzed with a CNN. Therefore, their findings support the presence of altered pattern in muscles with TrPs, but the physiological nature is unclear.

Hence, further studies are required to determine whether this spatial pattern is characteristic of TrPs and if the area of ARV overlaps with them. sEMG maps may eventually contribute to research on TrP localization, but robust validation studies are required.

### 4.3. Limitations and Risk of Overinterpretation

The findings presented in this review should be interpreted with caution due to several conceptual and methodological limitations within the current body of evidence. First, most included studies were cross-sectional and exploratory in nature, limiting the ability to infer causal relationships between TrPs and observed sEMG alterations. Although differences in electromyographic patterns were frequently identified between TrP-affected muscles and healthy controls, these changes cannot be considered specific biomarkers of TrPs, as similar alterations may occur in other painful musculoskeletal conditions, fatigue states, altered motor strategies, or pain-related neuromuscular adaptations. This limitation also relates to the broader distinction between chronic primary and chronic secondary musculoskeletal pain. Because TrP-related symptoms may include motor, sensory, autonomic, and referred components, future studies should determine whether sEMG abnormalities reflect local muscle dysfunction or broader sensorimotor adaptations. In post-traumatic musculoskeletal pain and functional disability, sensory and neurophysiological alterations have been investigated using objective functional assessment methods, including quantitative sensory testing, current perception threshold testing, and electrophysiological evaluation [89,90,91]. Integrating sEMG with sensory, autonomic, kinematic, or imaging-based measures may therefore clarify its diagnostic relevance within broader functional assessment frameworks.

Second, the neurophysiological interpretation of sEMG findings remains indirect. sEMG reflects the summation of motor unit action potentials rather than direct motor endplate activity. Consequently, it is not possible to directly attribute observed signal changes to specific TrP mechanisms such as endplate dysfunction or spontaneous electrical activity. Some mechanistic explanations proposed in the literature therefore remain hypothetical and should not be interpreted as definitive evidence of TrP pathophysiology.

Additionally, substantial heterogeneity existed across studies regarding diagnostic criteria, electrode placement, recording protocols, contraction tasks, and signal-processing methods. Sample sizes were generally small, definitions of active and latent TrPs varied, and several studies lacked externally validated diagnostic thresholds. Signal normalization was absent or inconsistent across the included literature, which further limits comparability between studies and increases the risk of overestimating the consistency or clinical applicability of the reported findings. The apparent promise of time–frequency and HDsEMG with machine learning approaches should also be interpreted cautiously because evidence currently derives from a limited number of studies, largely conducted by the same research groups, small sample sizes, potential overfitting and without external validation.

An additional limitation concerns the clinical reference standard used to identify TrPs. The Delphi consensus by Fernández-de-las-Peñas and Dommerholt helped standardize TrP diagnosis around three main criteria: a taut band, a hypersensitive spot, and referred pain [2]. However, some included studies were conducted before this consensus or did not fully follow this framework. TrP identification still relies mainly on palpation and symptom reproduction, both of which depend on the examiner and the patient. This may introduce misclassification bias, especially when latent TrPs are not excluded from control groups or when broad MPS diagnoses are used instead of point-specific TrP classification. Comparators also varied across studies. These included healthy controls, active vs. latent TrPs comparisons, affected vs. unaffected muscles, clinical comparison groups, and repeated within-session conditions. Therefore, the findings should be interpreted as study-specific contrasts rather than equivalent diagnostic comparisons. Technical sEMG heterogeneity was identified as an important feature of the evidence base. Differences in electrode placement, contraction tasks, acquisition settings, preprocessing, normalization, and outcome measures were closely linked with differences in anatomical region and study design. Therefore, in line with the scoping review approach, studies were organized by sEMG analysis domains to identify patterns, gaps, and areas with the greatest potential for further development, rather than by isolated acquisition parameters.

Several limitations of the review process should also be acknowledged. Although the search covered five major databases and was updated before manuscript submission, eligibility was restricted to English-language full-text publications, which may have led to the exclusion of relevant non-English, unpublished, or otherwise inaccessible studies. Grey literature was not systematically searched, and authors of primary studies were not contacted for additional information. Therefore, unclear or missing methodological details were treated as not reported. In addition, the developed review protocol was not registered a priori, but is available in Supplementary Materials (File S2: Review Protocol).

For these reasons, current evidence should be viewed as exploratory and hypothesis-generating rather than confirmatory. Further standardized and methodologically robust studies are necessary before specific sEMG patterns can be considered clinically reliable markers of TrP-related muscle dysfunction.

## 5. Conclusions

In conclusion, sEMG may be useful as a non-invasive complementary tool for functional assessment and monitoring of muscles affected by TrPs, but current evidence is insufficient for standalone diagnosis or clinically reliable classification of TrPs. Time–frequency analysis remains a promising domain, particularly MSWEV-based approaches for MPS-related muscle activation patterns and STFT/PSD approaches in experimentally provoked referred pain paradigms. HD-sEMG approaches and machine learning-based classification may provide complementary information. However, these findings remain preliminary and should not be generalized beyond the specific protocols in which they were observed. Techniques based solely on amplitude or conventional frequency analysis present important methodological limitations and may reflect nonspecific muscular changes rather than alterations directly related to TrPs. Although spatial analysis may provide relevant information about altered muscle activation patterns, robust and standardized studies are still needed to determine its clinically meaningful diagnostic relevance. Future research should report sensitivity, specificity, AUC, cut-off values, test–retest reliability, standardized electrode placement and normalization procedures, and external validation before clinically applicable diagnostic criteria can be proposed.

## Figures and Tables

**Table 1:** Search strategy in databases.

Database	Strategy	First Search	Second Search
PubMed	(“electromyography” [tiab] OR “EMG” [tiab]) AND (“Myofascial Pain Syndromes” [MeSH Terms] OR “myofascial pain syndrome” [tiab] OR “myofascial pain” [tiab] OR “trigger point” [tiab] OR “trigger points” [tiab])	381	22
Scopus	TITLE-ABS-KEY ((“electromyography” OR “EMG”) AND (“myofascial pain” OR “myofascial pain syndrome” OR “central sensitization” OR “trigger points” OR “trigger point”)) AND (LIMIT-TO (LANGUAGE, “English”)) AND (LIMIT-TO (EXACTKEYWORD, “Article”)) AND (LIMIT-TO (SRCTYPE, “j”)) AND (LIMIT-TO (DOCTYPE, “ar”))	532	33
Web of Science	TS = ((“electromyography” OR “EMG”) AND (“myofascial pain” OR “myofascial pain syndrome” OR “trigger points” OR “trigger point”))	354	31
CINAHL	(“Electromyography” OR “electromyography” OR “EMG”) AND (“Myofascial Pain Syndromes” OR “myofascial pain” OR “myofascial pain syndrome” OR “trigger point” OR “trigger points”)	854	83
Cochrane	(“electromyography”):kw AND ((“myofascial pain”):kw OR (“myofascial pain syndrome”):kw OR (“trigger points”):kw OR(“central sensitization”):kw OR (“trigger points”):kw)	285	46

First search—search conducted between 20 April and 25 April 2024; second search—update search conducted on 12 May 2026.

**Table 2:** Eligibility criteria.

Area	Inclusion Criteria	Exclusion Criteria
Scope	sEMG assessment of muscles with TrPs, MPS, or TrP-related referred pain.	nEMG only; no extractable sEMG data; animal studies; pediatric populations.
Population	Adults with active or latent TrPs, MPS.	Neurological, systemic, or primary muscle diseases if TrP-related data could not be separated.
Comparator/reference condition	Healthy/asymptomatic controls, clinical comparison groups, active vs. latent TrPs, TrP vs. no-TrP sites, affected vs. unaffected muscles, or repeated within-session conditions.	No comparator or reference condition allowing interpretation of TrP-related sEMG findings.
Study design	Observational, cross-sectional, case–control, diagnostic accuracy, and experimental/provocation studies.	Treatment efficacy studies where sEMG was used only to evaluate therapeutic response.
Language	English.	Other.
Availability	Full-text publications required for data extraction.	Full text not available for data extraction.

sEMG—surface electromyography; nEMG—needle electromyography; TrP(s)—trigger point(s); MPS—myofascial pain syndrome.

**Table 3:** Characteristics of included studies.

Authors	Study Design	Aim of the Study	Investigated Region	Experimental Group	Comparator/Reference	Diagnostic Criteria for TrPs/Myofascial Pain	TrPs (Active/Latent)
Zieliński et al., 2021 [23]	Cross-sectional	To analyze electromyographic patterns of masticatory muscles in relation to active trapezius TrPs and TMD.	Upper back, orofacial	Active trapezius TrPs: *n* = 60 (M:18; W:42), age 23 ± 3 years. TMD: *n* = 47 (M:14; W:33); age 33 ± 1 years.	Healthy controls without TMD and active/latent TrPs in head/neck muscles: *n* = 60 (M:18; W:42); age 23 ± 3 years.	Taut band + hyperirritable spot + referred pain. Active TrPs: reproduction of pain symptoms.	active
Manfredini et al., 2011 [49]	Cross-sectional	To assess the diagnostic accuracy of commercially available sEMG and kinesiography devices for myofascial pain of jaw muscles.	Orofacial region	Patients with MFP of jaw muscles diagnosed according to RDC/TMD Axis I: *n* = 36 (M:12; W:24); age 34 ± 9 years.	Age- and sex-matched TMD-free asymptomatic subjects: *n* = 36 (M:12; W:24).	RDC/TMD Axis I criteria, including jaw muscle pain, tenderness, and limited jaw movement.	not specified
Wytrążek et al., 2015 [24]	Cross-sectional	To assess TrPs and evaluate palpation, algometry, and sEMG for detection and differentiation of TrP types in young volunteers.	Neck, shoulder girdle	Participants with active/latent TrPs with referred pain or TrPs without referred pain: *n* = 70 (M:22; W:48); age 20.6 years.	Subgroup/no-TrPs comparison.	Taut band + hyperirritable spot + referred pain. Active TrPs: reproduction of pain symptoms.	active and latent
Lisiński and Huber, 2017 [25]	Cross-sectional	To determine symptoms of muscle dysfunction in patients with MPS, disc-root conflict, and degenerative cervical spine disease.	Neck, shoulder girdle, upper extremity muscles	MPS: *n* = 60 (W:35; M:25); age 40.5 ± 5.5 years. Disc-root conflict: *n* = 60 (W:19; M:41); age 52.2 ± 7.7 years. Degenerative spine disease: *n* = 60 (W:27; M:33); age 60.3 ± 9.1 years.	Healthy control group: *n* = 30 (W:18; M:12); age 48.6 ± 12.3 years.	Presence of active TrPs (0—no reaction; 1—tenderness). Criteria not fully specified in the extracted data.	active
Baraja-Vegas et al., 2019 [50]	Cross-sectional/provocation study	To evaluate EMG peak activity during local twitch responses and resting EMG before and after dry needling of latent TrPs in gastrocnemius medialis.	Lower extremity	Healthy males with latent TrPs in the gastrocnemius medial head: *n* = 20; age 25.5 ± 5 years.	Within-participant repeated measures: pre-DN rest, successive LTRs during DN, and post-DN rest.	Taut band + hyperirritable spot + local twitch response of the taut.	latent
Gemmell and Bagust, 2009 [51]	Cross-sectional	To determine whether sEMG can differentiate upper trapezius muscles with active vs. latent TrPs.	Upper back	Participants with either active or latent TrPs: *n* = 12 (M:5; W:7); age 28.9 years.	Active vs. latent TrPs within the study; no healthy control group.	Taut band + hyperirritable spot + referred pain. Active TrPs: reproduction of pain symptoms.	active and latent
Wytrążek et al., 2011 [52]	Cross-sectional	To evaluate whether muscle pain influences muscle strength and motor unit activity, and whether sEMG recordings at rest and during maximal contraction differ between muscles with and without TrPs.	Neck, upper back, lower back, pelvic girdle	Patients with nonspecific chronic cervical and back pain: *n* = 30 (W:23; M:7); age 34–67 years.	Healthy volunteers: *n* = 30 (W:14; M:16); age 19–54 years.	Taut band + hyperirritable spot + local twitch response of the taut band with palpation + pain reproduction.	active
Rahmati et al., 2026 [53]	Controlled observational study	To compare shoulder muscle activation patterns and kinematic parameters, particularly SHR, between individuals with upper trapezius TrPs and healthy controls during shoulder abduction.	Upper back	Active upper trapezius TrPs: *n* = 15 (M:3; W:12); age 29.4 ± 4.8 years.	Asymptomatic controls no neck/shoulder pain or palpable TrPs: *n* = 13 (M:3; W:10); age 28.5 ± 6.07 years.	Taut band + hyperirritable spot + referred pain and recognition of pain, LTR.	active
Seong Hun Yu and Hyun Jin Kim, 2015 [54]	Cross-sectional	Comparison of electrophysiological features of healthy vs. latent and active TrPs muscles to support MPS diagnosis.	Upper back	Active TrPs: *n* = 30, age 23.27 ± 4.21 years. Latent TrPs: *n* = 30; age 23.74 ± 6.47 years; sex not reported.	No pain or tenderness: *n* = 30; age 24.17 ± 3.14 years; sex not reported.	Active TrPs: pain at rest, taut bands, hyperirritable spot, referred pain, local twitch response, weakness. Latent TrPs: tenderness on stimulation without MPS diagnosis.	active and latent
Lin et al., 2018 [27]	Secondary analysis of cross-sectional study	To validate an MSWEV model combined with machine learning techniques to classify and differentiate sEMG patterns between MPS patients and healthy controls.	Upper back	MPS patients: *n* = 26 (W:22; M:4); age 35.96 ± 9.06 years.	Healthy controls: *n* = 30 (W:27; M:3); age 39.5 ± 9.00 years.	Taut band + pain persisting for more than 6 months + hyperirritable spot.	not specified
Konieczny et al., 2025 [26]	Experimental/provocation study	To assess electromyographic activity in thigh muscles in response to noxious stimulation of the gluteus minimus with elicited TrP-related referred pain.	Lower limb, pelvic girdle	Participants with elicited referred pain confirmed with amplified vasodilatation within the referred pain zone: *n* = 13; age 18.5 ± 1.5 years; sex not reported.	Participants without elicited referred pain: *n* = 15; age 19.5 ± 1.6 years; sex not reported.	Hyperirritable spot + referred pain.	latent
Jiang et al., 2013 [55]	Cross-sectional	To identify neuromuscular activation differences between patients with MPS and healthy individuals by applying MSWEV analysis on sEMG signals.	Upper back	MPS patients: *n* = 26 (W:22; M:4); age 35.96 ± 9.06 years.	Healthy controls: *n* = 30 (W:27; M:3); age 39.5 ± 9.00 years.	Taut band + pain persisting for more than 6 months + hyperirritable spot.	not specified
Barbero et al., 2013 [40]	Cross-sectional	To describe the location of TrPs and IZs in the upper trapezius muscle.	Upper back	Subjects with at least one TrP in the right upper trapezius muscle and neck pain involving the upper trapezius region within the previous 2 weeks: *n* = 24 (18 active TrPs, 6 latent TrPs); age and sex not reported.	Pain-free subjects with latent TrPs in the right upper trapezius muscle: *n* = 24; age and sex not reported.	Active TrP: taut band in the upper trapezius and at least one of the following: hyperirritable spot, reproduction of the patient’s pain, or referred pain upon pressure. Latent TrP: taut band and spot tenderness without pain reproduction.	active and latent
Barbero et al., 2016 [28]	Cross-sectional	To evaluate EMG amplitude distribution and peak activity location in the upper trapezius during shoulder elevation in subjects with and without TrPs, and compare peak locations to TrP sites.	Upper back	Subjects with at least one clinically relevant TrP in the right upper trapezius muscle and reported pain over the upper trapezius muscle in the last 2 weeks: *n* = 13 (W:7; M:6); age 22.8 ± 3.5 years.	Asymptomatic participants: *n* = 12 (W:5; M:7); age 21.8 ± 1.4 years.	Taut band + hyperirritable spot + referred pain and recognition of pain.	active
Lu et al., 2026 [56]	Prospective proof-of-concept classification study	To test whether upper-trapezius sEMG contraction signals contain discriminative information for classifying participants with TrPs versus healthy controls using a 1D CNN.	Upper back	TrPs: *n* = 13; Table 1 reports: latent *n* = 11 (M:5, W:6); age 33.7 ± 11.3 years. active *n* = 2 (M:2, W:0); age 25.0 ± 1.4 years. Note: the article later refers to latent *n* = 9 in sensitivity-analysis text.	Healthy controls without TrPs: *n* = 9; M:6, W:3; age 29.9 ± 13.6 years.	Classified by experienced pain specialist using manual palpation.	active/latent

n—group size; W—women; M—men; TrPs—trigger points; TMD—temporomandibular disorder; sEMG—surface electromyography; RDC/TMD—Research Diagnostic Criteria for Temporomandibular Disorders; MPS—myofascial pain syndrome; MSWEV—multi-scale wavelet energy variation; IZs—innervation zones.

**Table 4:** sEMG properties in the included studies.

Authors	sEMG Equipment	Electrodes	Signal Normalization	EMG Signal Collection Properties
**Amplitude domain analysis**				
Zieliński et al., 2021 [23]	BioEMG III™ (BioResearch Associates, Inc., Mequon, WI, USA)	Bipolar electrodes on temporalis anterior and masseter muscles according to SENIAM (temporalis anterior, masseter muscles).	Not applied	Amplification and filtering: Digital NoiseBuster filter (linear scale, 99% noise reduction).
Manfredini et al., 2011 [49]	K6 Diagnostic System^®^ (Myotronics Inc., Seattle, WA, USA)	Bipolar surface electrodes (Duotrode; Myotronics Inc., Seattle, WA, USA) placed bilaterally over the masseter and temporalis muscles.	Not applied	Not reported in the article.
Wytrążek et al., 2015 [24]	Keypoint System (Medtronic A/S, Skovlunde, Denmark)	Bipolar electrodes placed over muscle bellies and distal tendons (Trapezius, Sternocleidomastoid, Deltoid, Infraspinatus).	Not applied	Band-pass: 20–10,000 Hz; acquisition settings reported as 10–400 Hz; amplification: MVC 1000 µV/D, rest 20 µV/D; time-base: 80 ms/D.
Lisiński & Huber, 2017 [25]	Keypoint system (Medtronic A/S, Skovlunde, Denmark)	Bipolar electrodes placed over muscle bellies and distal tendons (Trapezius, Sternocleidomastoid, biceps brachii, abductor pollicis brevis).	Not applied	Band-pass: 20–10,000 Hz; acquisition settings reported as 10–400 Hz; amplification: MVC 1000 µV/D, rest 20 µV/D; time-base: 80 ms/D.
Baraja-Vegas et al., 2019 [50]	BTS FREEMG 300 System (BTS Bioengineering, Quincy, MA, USA)	Electrodes (Lessa 99.830.17, BTS FREEMG 300) were positioned 20 mm apart, one over and one under the TrP area, and firmly fixed with adhesive tape bilaterally over the medial gastrocnemius muscle belly.	Not applied	Band-width: 10–450 Hz; band-pass: 20–400 Hz (second order Butterworth).
Gemmell and Bagust, 2009 [51]	BioPac MP150 system (BioPac, Goleta, CA, USA)	Three disposable pre-gelled electrodes per shoulder, placed parallel to upper trapezius muscle fibres.	RMS normalized to mean shrug amplitude	Band-pass filter: 10–500 Hz; high-pass filter: 50 Hz.
Wytrążek et al., 2011 [52]	Keypoint System (Medtronic A/S, Denmark)	Standard bipolar electrodes placed over the muscle belly and tendon for sEMG (Upper Trapezius, Gluteus medius, Tensor fasciae latae, Lumbar erector spinae).	Not applied	Band-pass filter: 10 Hz to 10 kHz; time base: 80 ms/D; amplification range: 20 µV to 1000 µV/D.
Rahmati et al., 2026 [53]	Wireless EMG system (Aktos, Mayon Inc., Switzerland)	Surface electrodes over upper, middle and lower trapezius, serratus anterior, anterior/middle/posterior deltoid and supraspinatus according to SENIAM.	Normalized to MVC EMG value for each muscle	Sampling rate 1200 Hz. Band-pass 10–500 Hz, third-order Butterworth; 50 Hz notch; rectification; 100 ms smoothing windows; input impedance > 100 MΩ; CMRR > 100 dB; gain 1000.
**Frequency domain analysis**				
Yu and Kim, 2015 [54]	AcqKnowledge 3.8.1 (Biopac Systems, Goleta, CA, USA); acquisition device not reported	Upper trapezius muscle.	Not applied	Sampling rate 1000 Hz; band-pass filter 20–450 Hz.
**Time–frequency analyses**				
Lin et al., 2018 [27]	MA-411 electrodes; acquisition device not specified	Electrodes placed bilaterally on the upper back along the taut band (trapezius muscle).	Not applied	Sampling rate 5000 Hz; band-pass 20–3000 Hz; wavelet filtering to reduce noise.
Konieczny et al., 2025 [26]	NORAXON DTS (Noraxon USA, Inc., Scottsdale, AZ, USA)	Sensors and electrodes were placed according to the SENIAM method (gluteus medius, gluteus maximus, vastus lateralis, rectus femoris, vastus medialis, adductor longus, biceps femoris and semitendinosus).	Not applied	Low-pass filter: 500 Hz; sampling rate: 1500 Hz.
**Time–frequency, amplitude, and frequency domain analysis**				
Jiang et al., 2013 [55]	Device not specified; electrodes MA 411	Electrodes placed bilaterally on the upper back along the taut band (trapezius muscle).	Not applied	Sampling rate 5000 Hz; band-pass 20–3000 Hz; wavelet filtering to reduce noise.
**HDsEMG**				
Barbero et al., 2013 [40]	OT-Bioelettronica (Torino, Italy)	High-density surface EMG, monopolar configuration, 64-electrode grid (13 rows × 5 columns; electrode diameter 2 mm; inter-electrode distance 8 mm), placed over upper trapezius (medial to innervation zone).	Not applied	Fourth-order Butterworth noncausal filter (20–450 Hz); sampling rate: 2048 Hz.
Barbero et al., 2016 [28]	OT-Bioelettronica (Torino, Italy)	High-density surface EMG, monopolar configuration, 64-electrode grid (13 rows × 5 columns; electrode diameter 2 mm; inter-electrode distance 8 mm), placed over upper trapezius (medial to innervation zone).	Not applied	Fourth-order Butterworth noncausal filter (20–450 Hz); sampling frequency: 2048 Hz.
Lu et al., 2026 [56]	Saga 64 (TMSi, Oldenzaal, The Netherlands); 64-channel sEMG	64 electrodes placed over marked upper-trapezius TrP locations.	Z-score standardization after filtering,	Sampling rate 4000 Hz; fourth-order Butterworth band-pass filter 10–500 Hz; RMS rectification; smoothing; values above the 95th percentile removed as outliers; automated contraction-segment extraction.

sEMG—surface electromyography; EMG—electromyography; HDsEMG—high-density surface electromyography; SENIAM—Surface Electromyography for the Non-Invasive Assessment of Muscles; MVC—maximal voluntary contraction; RMS—root mean square; TrP—trigger point; CMRR—common-mode rejection ratio; Hz—hertz; kHz—kilohertz; µV/D—microvolts per division; dB—decibel.

**Table 5:** A summary of the outcomes of the included studies.

Authors	Trial Used to Extract Data	Results	Conclusion	Diagnostic Metrics Reported
**Amplitude domain analysis**				
Zieliński et al., 2021 [23]	At rest, maximal mouth opening, clenching.	At rest: Higher EMG amplitudes in the temporalis anterior for both TrPs and TMD in comparison to healthy subjects. Clenching: TrPs—increased TA activity, positive AcI, high MM activity; TMD—negative AcI, TA dominance, decreased MM activity, high TA MVC.	EMG patterns differentiate TrPs and TMD from healthy controls, but differences between TrPs and TMD need further investigation.	N/R
Manfredini et al., 2011 [49]	Rest and MVC/clenching tasks.	Rest: no significant differences in amplitude between patients and controls. MVC/clenching: significantly decreased amplitude in MPS compared with healthy controls.	sEMG and kinesiography devices were not reliable for diagnosing myofascial pain in individual patients due to high false-positive rates. EMG activity during clenching tasks was the only parameter with acceptable diagnostic accuracy.	Clenching: sensitivity 77.8–91.7%; specificity 76.7–86.7%. Resting EMG: sensitivity 43.5–52.2%; specificity 27.8–55.6%.
Wytrążek et al., 2015 [24]	Rest and MVC.	Rest: sEMG amplitude significantly higher in active and latent TrPs with/without referred pain compared with no-TrP sites. MVC: no significant changes.	Resting sEMG amplitude can confirm the presence of TrPs detected by palpation and algometry.	N/R
Lisiński & Huber, 2017 [25]	At rest, MVC.	At rest: Increased amplitude in sternocleidomastoid and trapezius muscles, primarily in MFP and similarly in disc-root conflict. MVC: Decreased amplitude for MFP.	Differentiation of neck pain origins requires combined clinical and neurophysiological testing, with trapezius (MFP) and abductor pollicis brevis muscles (nerve-root conflict) serving as key indicators.	N/R
Baraja-Vegas et al., 2019 [50]	At rest: five minutes before and after DN; during DN: successive LTRs within the same participants.	At rest: no changes in RMS before and after DN. During DN, the RMS peak amplitude of successive LTRs decreased compared with previous responses in the same participants with latent TrPs in the medial gastrocnemius.	The decrease in EMG peak muscle activity during successive LTRs may be related to decreased neural pool excitability in the spinal cord.	N/R
Gemmell and Bagust, 2009 [51]	Rest: 1 min before shrugs; MVC: shoulder shrugs for 3 s; post-shrug relaxation: three 1 min segments.	MVC: muscles with active TrPs exhibited lower RMS amplitude than those with latent TrPs, but the difference was not statistically significant. Post-shrug relaxation: resting activity increased compared with baseline for both latent and active TrPs.	Despite not reaching significance, sEMG showed potential for differentiating active from latent TrPs; further studies with larger samples are needed.	N/R
Wytrążek et al., 2011 [52]	Rest and MVC.	Rest: increased sEMG amplitude in muscles with TrPs. MVC: decreased sEMG amplitude in TrP-affected muscles.	Increased resting sEMG amplitude and reduced MVC sEMG amplitude were observed in muscles with TrPs; sEMG may support functional assessment but does not provide standalone diagnosis.	N/R
Rahmati et al., 2026 [53]	Dominant-side shoulder abduction from 0 degrees to full elevation; 3 repetitions; EMG from eight shoulder muscles.	Peak EMG amplitude, RMS, and onset timing did not differ between groups. Rise time and SHR were significantly higher in the TrP group, suggesting altered neuromuscular timing and shoulder coordination.	Amplitude/RMS measures alone did not discriminate active upper-trapezius TrPs from controls. Temporal EMG rise time and SHR may be more sensitive to subtle neuromuscular/kinematic adaptations.	N/R
**Frequency domain analysis**				
Yu and Kim, 2015 [54]	MVC	MDF and mean muscle fatigue index (MFI): significantly higher in the active TrPs group compared with healthy subjects.	MDF and MFI may support quantitative assessment of TrP-related muscle alterations, but these findings require further validation.	N/R
**Time–frequency analyses**				
Lin et al., 2018 [27]	A 5-beat action cycle (1 beat/s) was performed six times over 1 min: rest (beats 1–3), maximal bilateral arm lift (beat 4), and release (beat 5).	MSWEV-based machine learning features enabled classification of patients based on energy-pattern differences. These values reflect classification accuracy/consistency rather than diagnostic sensitivity/specificity.	Moderate classification performance suggests potential for using MSWEV graphs to characterize sEMG signal changes associated with TrPs/MPS, but this does not yet constitute validated diagnostic accuracy.	Classification accuracy: 77% using template matching and 60% using K-means clustering. Classification consistency between methods: 87% in the normal group and 93% in the MPS group.
Konieczny et al., 2025 [26]	10 min of recording during dry needling/provocation.	Higher PSD was observed in subjects with elicited referred pain; STFT showed altered spectral activity in muscles located within the referred pain area compared with those without elicited referred pain.	The observed motor phenomenon suggests potential for objectively assessing alterations associated with experimentally provoked TrP-related referred pain.	N/R
**Time–frequency, amplitude, and frequency domain analysis**				
Jiang et al., 2013 [55]	A 5-beat action cycle (1 beat/s) was performed six times over 1 min: rest (beats 1–3), maximal bilateral arm lift (beat 4), and release (beat 5).	MSWEV: Healthy controls showed relatively greater high-frequency energy; MPS patients showed relatively greater low-frequency energy. RMS: Patients with MPS showed earlier activation than controls. MDF: Significant group differences were reported at selected rest and contraction phases.	MSWEV was more informative than RMS and MDF in this study for detecting subtle neuromuscular differences between MPS and healthy controls.	MSWEV visual classification: sensitivity 53.85%, specificity 83.33%.
**HDsEMG**				
Barbero et al., 2013 [40]	Isometric contraction of the upper trapezius at 20% of maximum voluntary contraction.	IZs were located in a limited area in the second and third quadrants of the ACS, with no significant difference between active and latent TrP locations.	TrPs and IZs are located in well-defined areas of the upper trapezius muscle, with TrPs being situated proximally to the IZ but not overlapping.	N/R
Barbero et al., 2016 [28]	Shoulder elevation task: ramped contractions at 15% and 60% MVC, each lasting 60 s, analyzed in 1 s epochs.	Caudal shift (lower location) of peak EMG amplitude in subjects with TrPs compared to controls. No direct association between peak EMG amplitude and trigger point locations.	The presence of TrPs alters the spatial distribution of trapezius EMG activity, but peak EMG activity is not directly associated with TrP locations.	N/R
Lu et al., 2026 [56]	64-channel upper-trapezius sEMG during dumbbell shrugging; pre- and post-stretching contraction segments extracted automatically.	The 1D CNN classified TrP versus healthy participants using HD-sEMG contraction segments. The best subject-level performance was obtained for pre-intervention segments (ROC-AUC 0.940; accuracy 0.960). In latent-only analysis, the best subject-level model reached ROC-AUC 0.916 and accuracy 0.930.	sEMG contraction segments may contain discriminative information for classifying primarily latent upper-trapezius TrPs, but findings require validation in larger cohorts.	Full-group subject-level: pre-intervention: ROC-AUC 0.940 (95% CI 0.88–0.98), accuracy 0.960 (0.93–0.99), F1-score 0.960 (0.93–0.99), MCC 0.933 (0.88–0.99), Youden index 0.933 (0.88–0.99). Latent-only subject-level: combined pre/post: ROC-AUC 0.916 (0.86–0.96), accuracy 0.930 (0.88–0.97), F1-score 0.928 (0.87–0.97), MCC 0.882 (0.79–0.94), Youden index 0.887 (0.80–0.95).

MVC—maximum voluntary contraction; MM—masseter muscle; TA—temporalis anterior; AcI—Activity Index (relative muscle activity index from EMG signal); DN—dry needling; RMS—root mean square; LTR—local twitch response; MDF—median frequency; MFI—muscle fatigue index; PSD—power spectral density; TrPs—trigger points; TMD—temporomandibular disorder; sEMG—surface electromyography; MPS—myofascial pain syndrome; MSWEV—multi-scale wavelet energy variation; IZs—innervation zones; ACS—anatomical coordinate system; STFT—short-time Fourier transform; AUC—area under the curve; CNN—convolutional neural network; ROC-AUC—area under the receiver operating characteristic curve; SHR—scapulohumeral rhythm; N/R—not reported.

## Data Availability

The data presented in this study are available on request from the corresponding author.

## Supplementary Materials

The following supporting information can be downloaded at https://www.mdpi.com/article/10.3390/biomedicines14061406/s1, Supplementary File S1: Table S1: PRISMA-ScR checklist; Figure S1A–C: Critical Appraisal; Supplementary File S2: Review Protocol.

## Abbreviations

The following abbreviations are used in this manuscript:

AcI	Activity Index
ACS	anatomical coordinate system
ARV	average rectified value
CINAHL	Cumulative Index to Nursing and Allied Health Literature
DN	dry needling
ECG	electrocardiography/electrocardiographic
EMG	electromyography
ICD-11	International Classification of Diseases, 11th Revision
IZ/IZs	innervation zone(s)
LTR/LTRs	local twitch response(s)
MDF	median frequency
MFI	muscle fatigue index
MFP	myofascial pain
MG30	ICD-11 chronic primary pain category/code MG30
MM	masseter muscle
MPS	myofascial pain syndrome
MRI	magnetic resonance imaging
MSWEV	multi-scale wavelet energy variation
MVC	maximal voluntary contraction
N/A	not applicable
nEMG	needle electromyography
PCC	Population, Concept, Context
PRISMA-ScR	Preferred Reporting Items for Systematic Reviews and Meta-Analyses Extension for Scoping Reviews
PSD	power spectral density
RDC/TMD	Research Diagnostic Criteria for Temporomandibular Disorders
RMS	root mean square
sEMG	surface electromyography
SENIAM	Surface Electromyography for the Non-Invasive Assessment of Muscles
SP	SP test
STFT	short-time Fourier transform
TA	temporalis anterior
TMD	temporomandibular disorder
TrP/TrPs	trigger point(s)
US	ultrasonography
