# Supplementary material for: Diagnostic Utility of Surface Electromyography for Identifying Muscles Affected by Myofascial Trigger Points: A Scoping Review
Source: Biomedicines. 2026 Jun 22;14(6):1406. doi: 10.3390/biomedicines14061406 (PMC13297627; doi:10.3390/biomedicines14061406)
Supplement: Supplementary file 1 [file biomedicines-14-01406-s001.zip › Supplementary File S1.zip!/Figure S1A-C Critical Appraisal.pdf]

Figure S1A-C: Critical Appraisal

	A1	A2	A3	A4	A5	A6	A7	A8
Zielinski et al., 2021	Y	Y	Y	Y	U	U	Y	Y
Wytrazek et al., 2015	U	U	Y	Y	U	N	Y	Y
Lisinski & Huber, 2017	Y	Y	Y	Y	U	N	Y	Y
Gemmell & Bagust, 2009	U	Y	Y	Y	N	N	U	Y
Wytrazek et al., 2011	Y	U	Y	Y	U	N	Y	Y
Rahmati et al., 2026	Y	Y	Y	Y	U	U	Y	Y
Yu & Kim, 2015	U	U	Y	Y	N	N	Y	U
Barbero et al., 2013	Y	Y	Y	Y	U	N	Y	Y
Barbero et al., 2016	Y	Y	Y	Y	U	N	Y	Y

Repsonse key: Y = Yes; N = No; U = Unclear; NA = Not applicable

Checklist items:

A1. Were the criteria for inclusion in the sample clearly defined?
A2. Were objective, standard criteria used for measurement of the condition?
A3. Was the exposure measured in a valid and reliable way?
A4. Were the outcomes measured in a valid and reliable way?
A5. Were confounding factors identified?
A6. Were strategies to deal with confounding factors stated?
A7. Was appropriate statistical analysis used?
A8. Were the study subjects and the setting described in detail?

Figure S1A. JBI critical appraisal: analytical cross-sectional studies

Barker TH, Hasanoff S, Aromataris E, Stone JC, Leonardi-Bee J, Sears K, et al. The revised JBI critical appraisal tool for the assessment of risk of bias for analytical cross-sectional studies. JBI Evid Synth. 2026;24(3):401-8.

	Q1	Q2	Q3	Q4	Q5	Q6	Q7	Q8	Q9
Konieczny et al., 2025	Y	Y	N	U	U	Y	Y	NA	Y
Baraja-Vegas et al., 2019	Y	N	NA	NA	Y	Y	Y	NA	Y

Repsonse key: Y = Yes; N = No; U = Unclear; NA = Not applicable

Checklist items:

Q1. It is clear in the study what is the "cause" and what is the "effect" (ie, there is no confusion about which variable comes first)?
Q2. Was there a control group?
Q3. Were participants included in any comparisons similar?
Q4. Were the participants included in any comparisons receiving similar treatment/care, other than the exposure or intervention of interest?
Q5. Were there multiple measurements of the outcome, both pre and post the intervention/exposure?
Q6. Were the outcomes of participants included in any comparisons measured in the same way?
Q7. Were outcomes measured in a reliable way
Q8. Was follow-up complete and if not, were differences between groups in terms of their follow-up adequately described and analyzed?
Q9. Was appropriate statistical analysis used?

Figure S1B. JBI critical appraisal: quasi-experimental studies

Barker, Timothy H.; Habibi, Nahal; Aromataris, Edoardo; Stone, Jennifer C.; Leonardi-Bee, Jo; Sears, Kim; et al. The revised JBI critical appraisal tool for the assessment of risk of bias for quasi-experimental studies. JBI Evidence Synthesis 22(3):p 378-388, March 2024. | DOI: 10.11124/JBIES-23-00268

	D1	D2	D3	D4	D5	D6	D7	D8	D9	D10
Manfredini et al., 2011	Y	N	Y	Y	U	Y	Y	Y	Y	Y
Jiang et al., 2013	N	N	U	N	N	U	Y	Y	Y	Y
Lin et al., 2018	N	N	U	U	N	U	Y	Y	Y	Y
Lu et al., 2026	N	N	U	U	N	U	Y	Y	Y	N

Repsonse key: Y = Yes; N = No; U = Unclear; NA = Not applicable

Checklist items:

D1. Was a consecutive or random sample of patients enrolled?
D2. Was a case control design avoided?
D3. Did the study avoid inappropriate exclusions?
D4. Were the index test results interpreted without knowledge of the results of the reference standard?
D5. If a threshold was used, was it pre-specified?
D6. Is the reference standard likely to correctly classify the target condition?
D7. Were the reference standard results interpreted without knowledge of the results of the index test
D8. Was there an appropriate interval between index test and reference standard?
D9. Did all patients receive the same reference standard?
D10. Were all patients included in the analysis?

Figure S1C. JBI critical appraisal: diagnostic test accuracy study

Campbell JM, Klugar M, Ding S, Carmody DP, Hakonsen SJ, Jadotte YT, White S, Munn Z. Chapter 9: Diagnostic test accuracy systematic reviews. In: Aromataris E, Munn Z (Editors). JBI Manual for Evidence Synthesis. JBI, 2020
