# Supplementary material for: Diagnostic Utility of Surface Electromyography for Identifying Muscles Affected by Myofascial Trigger Points: A Scoping Review
Source: Biomedicines. 2026 Jun 22;14(6):1406. doi: 10.3390/biomedicines14061406 (PMC13297627; doi:10.3390/biomedicines14061406)
Supplement: Supplementary file 1 [file biomedicines-14-01406-s001.zip › Supplementary File S2.zip!/File S2 Review Protocol.pdf]

Supplementary File S2

Protocol of Scoping Review: *“Diagnostic Utility of Surface Electromyography for Identifying Muscles Affected by Myofascial Trigger Points: A Scoping Review”*

This protocol defines the planned methods for conducting a scoping review on the diagnostic utility and potential clinical relevance of surface electromyography (sEMG) for identifying muscles affected by myofascial trigger points (TrPs), myofascial pain syndrome (MPS), or TrPs-related referred pain. The review will be conducted using the Population-Concept-Context (PCC) framework and reported in accordance with PRISMA-ScR.

1. Objective
   The objective of this scoping review is to map and synthesize available evidence on how sEMG has been used to assess TrPs-related neuromuscular alterations, with attention to signal-analysis domains, comparator/reference conditions, diagnostic or classification metrics, and methodological limitations relevant to future clinical research.

2. Research question

   I. What sEMG parameters have been used to assess muscles affected by TrPs, MPS, or TrPs-related referred pain?
   II. Which sEMG signal-analysis domains have been investigated in this context?
   III. What evidence exists regarding diagnostic or classification performance of sEMG-based approaches for TrPs-related muscle alterations?
   IV. What methodological, clinical-reference, comparator, and technical limitations affect interpretation of current sEMG findings in TrPs-related research?

3. PCC Framework

PCC element	Definition used in this review	Operationalization
Population	Adults with active or latent myofascial trigger points, myofascial pain syndrome, or TrPs-related referred pain.	Adult human participants; symptomatic or asymptomatic TrPs; active or latent TrPs; MPS populations.
Concept	Assessment of muscle properties or neuromuscular alterations using surface electromyography.	Amplitude-, frequency-, time-frequency-, spatial/high-density-, and machine-learning-supported sEMG parameters; diagnostic/classification metrics when available.
Context	Clinical, laboratory, observational, cross-sectional, controlled observational, diagnostic/classification, or experimental/provocation research.	Studies in which sEMG was used to evaluate muscles affected by TrPs, MPS, or TrPs-related referred pain under a meaningful comparator or reference condition.

4. Eligibility criteria

Area	Inclusion criteria	Exclusion criteria
Scope	sEMG assessment of muscles with TrPs, MPS, or TrPs-related referred pain.	Needle EMG only; no extractable sEMG data; animal studies; paediatric populations.
Population	Adults with active or latent TrPs, MPS, or TrPs-related referred pain.	Neurological, systemic, or primary muscle diseases if TrPs-related data could not be separated.
Comparator/reference condition	Healthy/asymptomatic controls, clinical comparison groups, active versus latent TrPs, TrP versus no-TrP sites/subgroups, affected versus unaffected muscles, provoked referred-pain/no-referred-pain conditions, or repeated within-session conditions.	No comparator or reference condition allowing interpretation of TrPs-related sEMG findings.
Study design	Observational, cross-sectional, controlled observational, case-control, diagnostic/classification, and experimental/provocation studies.	Treatment efficacy studies in which sEMG was used only to evaluate therapeutic response.
Language	English.	Other languages.
Availability	Full-text publications required for data extraction.	Full text unavailable for data extraction.

5. Information sources and search strategy

The following databases will be searched: PubMed, Scopus, Web of Science, CINAHL, and Cochrane. Search terms will combine electromyography/sEMG terms with myofascial pain, myofascial pain syndrome, trigger point, and trigger points terms. The PubMed strategy will be developed first using MeSH terms and title/abstract terms, then adapted to the syntax of the remaining databases. Additional potentially relevant records may be identified using citation chasing or similar-article functions when appropriate.

Database	Core search structure
PubMed	(electromyography OR EMG) AND (Myofascial Pain Syndromes OR myofascial pain syndrome OR myofascial pain OR trigger point OR trigger points)
Scopus	TITLE-ABS-KEY terms combining electromyography/EMG with myofascial pain/MPS/trigger point terms; limited to English journal articles.

Web of Science	TS terms combining electromyography/EMG with myofascial pain/MPS/trigger point terms.
CINAHL	Electromyography/EMG combined with Myofascial Pain Syndromes/myofascial pain/MPS/trigger point terms.
Cochrane	Keyword search combining electromyography with myofascial pain/MPS/trigger point terms.

6. Record management and study selection
   Search results will be imported into Rayyan.ai or equivalent review-management software. Duplicate records will be identified automatically and verified manually. Titles and abstracts will be screened independently by at least two reviewers according to predefined eligibility criteria. Full-text articles will then be assessed independently. Disagreements will be resolved through discussion, and unresolved disagreements will be adjudicated by a supervising author.

7. Data extraction plan
   Data will be extracted using a standardized data-extraction form. Extracted variables will include study characteristics, participant demographics, anatomical region, TrPs/MPS diagnostic criteria, active/latent TrP classification, comparator/reference condition, sEMG equipment, electrode placement, signal acquisition parameters, signal processing, normalization, analytical domain, outcome measures, diagnostic/classification metrics, and main findings.
   .

General characteristics	Authors, year, study design, aim, anatomical region, sample size, sex and age distribution, experimental group, comparator/reference condition.
Clinical reference standard	TrPs/MPS diagnostic criteria, active/latent TrP classification, symptom reproduction, referred pain, local twitch response or other clinical criteria when applicable.
Technical sEMG characteristics	Equipment, electrode type and placement, SENIAM use where applicable, filtering, sampling rate, signal processing, normalization, contraction task or recording condition.
sEMG outcomes	Amplitude, frequency, time-frequency, spatial/high-density, and machine-learning features; reported diagnostic or classification metrics including sensitivity, specificity, accuracy and others.

8. Data synthesis and charting
   Findings will be charted descriptively and synthesized narratively. Studies will be grouped according to sEMG signal-analysis domain: amplitude-domain, frequency-domain, time-frequency-domain, high-density/spatial sEMG, and machine-learning-supported approaches. Comparator/reference conditions

will be mapped explicitly and interpreted as study-specific contrasts rather than pooled as equivalent diagnostic evidence. Meta-analysis is not planned because heterogeneity is expected in populations, TrPs diagnostic criteria, comparator/reference conditions, anatomical regions, sEMG acquisition protocols, signal-processing approaches, and outcome measures.

9. Critical appraisal

Critical appraisal will not be used as an exclusion criterion because the objective of the review is evidence mapping.

10. Planned reporting outputs

1) PRISMA-ScR flow diagram describing record identification, screening, eligibility, and inclusion.
2) Tables summarizing study characteristics, diagnostic criteria, comparator/reference conditions, sEMG protocols, and sEMG outcomes.
3) Narrative synthesis emphasizing exploratory, complementary, and functional interpretation of sEMG findings.

11. Interpretation principles

The review will avoid treating group-level physiological differences as validated individual-level diagnostic performance. Findings will be interpreted as evidence regarding functional muscle alterations, exploratory classification approaches, and potential diagnostic relevance only when supported by sensitivity, specificity, ROC-AUC, or comparable performance metrics. Evidence will not be considered sufficient for standalone sEMG-based diagnosis unless validated diagnostic performance and external reproducibility are demonstrated.
